# Predictors of Caregiver Burden among Mothers of Children with Chronic Conditions

**DOI:** 10.3390/children4050039

**Published:** 2017-05-16

**Authors:** Karina Javalkar, Eniko Rak, Alexandra Phillips, Cara Haberman, Maria Ferris, Miranda Van Tilburg

**Affiliations:** 1 University of North Carolina School of Medicine, Chapel Hill, NC 27599, USA; 2 Department of Allied Health Sciences, University of North Carolina School of Medicine, Chapel Hill, NC 27599, USA; eniko_rak@med.unc.edu; 3 University of North Carolina Kidney Center, School of Medicine, Chapel Hill, NC 27599, USA; alphi@live.unc.edu (A.P.); maria_ferris@med.unc.edu (M.F.); 4 Wake Forest Baptist Medical Center, Winston-Salem, NC 27157, USA; chaberma@wakehealth.edu; 5 Department of Clinical Research, College of Pharmacy and Health Sciences, Campbell University, Buies Creek, NC 27506, USA; miranda_van_tilburg@med.unc.edu; 6 Department of Gastroenterology and Hepatology, School of Medicine, The University of North Carolina, Chapel Hill, NC 27599, USA; 7 Department of Social Work, University of Washington, Seattle, WA 98105, USA

**Keywords:** caregivers, disabilities, disease management, chronic conditions, health utilization

## Abstract

Objective: The complex medical regimens of children and adolescents with chronic conditions can have a significant impact on families and households. Caregivers may experience burden, which can lead to negative health consequences and poor quality of life. The objective of this study was to determine child-related predictors and risk factors for caregiver burden among parents of children with chronic conditions. Methods: We distributed an institutional review board (IRB)-approved, online cross-sectional survey to parents of children who attended the Victory Junction therapeutic camp. Parents provided information on child demographics, disease characteristics, and healthcare utilization. Parents also answered the adapted Zarit Burden Interview, which measured caregiver burden. Children completed scales about self-management and self-efficacy. Linear regression analyses determined how children’s disease characteristics, health utilization, and self-management skills were associated with caregiver burden. Results: We enrolled 150 mother-child dyads. The mean age of child participants was 12.23 years (±2.5), with an age range of 6 to 16 years. It was determined that children’s number of medicines and injections (β = 0.161, *p* = 0.047), a diagnosis of attention-deficit/hyperactivity disorder (ADHD) in addition to the primary medical condition (β = 0.216, *p* = 0.008), frequent visits with a primary care provider (PCP) (β = 0.209, *p* = 0.026) and emergency room (ER) visits (β = 0.197, *p* = 0.038), and lower child self-efficacy (β = −0.241, *p* = 0.041) were predictors of increased caregiver burden. Conclusions: We identified risk factors for caregiver burden among mothers. Future studies should explore additional child-related characteristics as they relate to caregiver burden, and should determine if interventions for mothers of children with chronic conditions can lead to positive outcomes.

## 1. Introduction

According to the National Survey of Children with Special Health Care Needs, there are 11.2 million children with chronic conditions and disabilities in need of special healthcare in the United States, and 1 in 3 have multiple comorbidities that significantly impact their health and functioning [1]. Additionally, epidemiologic studies have found that the incidence of chronic conditions such inflammatory bowel diseases and kidney disease among youth has increased over time [2,3].

Children and adolescents (youth) with chronic conditions have a medical regimen to follow that most commonly involves taking medications, attending medical appointments, or performing procedures such as injections, catheterization, or home dialysis. In addition, with various conditions such as those with physical disabilities, there is an increased need for assistance with basic activities of daily living (e.g., self-care or mobility). Early in life, children rely on caregivers for support and management of these tasks [4]. Caregivers of children with a chronic illness may experience poor sleep quality [5], family conflict [6], anxiety, depression [7], financial stress [8], and lower quality of life [9]. The 2011–2012 National Survey of Children’s Health found that only 56.7% of mothers and 62% of fathers reported excellent or very good physical and emotional health. For parents of children with chronic conditions, these numbers may be even lower. Poor caregiver well-being in turn may have a negative impact on the child and the quality of care provided [10]. Families and households are consequently impacted by having a child with a chronic condition [11]. Therefore, it is important to determine predictors of caregiver burden in order to identify risk factors and targets for interventions.

Predictors of caregiver burden have been explored among caregivers of the elderly population [12,13]. In the pediatric population, research is more limited. Mothers are most often the caregivers for children. Inappropriate behavior of the child [14] and caregiving demands have been identified as predictors of stress [15]. Family circumstances, such as single marital status or the ill child being an only child were also found to predict caregiver burden [16]. The majority of studies identified in a recent meta-analysis on parenting stress focused only on predictors related to a single condition, and most studies focused on asthma, diabetes, cancer, or cystic fibrosis [17]. The few studies that explored multiple conditions explored a narrow range of conditions (two to four conditions). Therefore, there has been a lack of identification of predictors of caregiver burden across a broad spectrum of conditions, including rare conditions.

Most studies examining predictors of caregiver burden have focused on caregiver-related characteristics such as education, employment, or caregiving duties. However, the literature is lacking in an understanding of child-related characteristics that may predict caregiver burden, such as child’s personality and behavior, demographic characteristics, or condition-related characteristics. In this study, we explored children’s diagnosis-related characteristics, healthcare utilization, and self-management as predictors of caregiver burden. We hypothesized that caregivers of children with attention-deficit/hyperactivity disorder (ADHD) as a comorbidity, greater numbers of medicines, greater emergency room (ER) utilization, lower primary care utilization, and poorer self-management skills experience higher levels of caregiver burden.

## 2. Methods

### 2.1. Study Design and Data Collection

Our cross-sectional study utilized a web-based survey distributed electronically on the survey engine Qualtrics™ (Provo, UT, USA) to potential participants. All participants who attended Victory Junction, a therapeutic summer camp in Randleman, North Carolina (USA), and their parents/guardians were invited to complete the survey. Institutional Review Board approval was obtained, and data was collected between May and August 2014.

### 2.2. Sample

A total of 781 invitations to participate were sent through email, and we received 160 responses. Campers were between the ages of 6 and 16 years, and had at least one chronic illness or disability. The survey was administered in English, and thus those unable to complete the survey in English were ineligible to participate.

### 2.3. Measures

Caregivers (parents/guardians) provided information on their child’s race, gender, age, primary diagnosis, and their child’s age at diagnosis. We calculated percentage of life with the disease as: (Age − Age of diagnosis)/Age). We coded each diagnosis as either a physical disability or a non-physical disability, based on the child’s primary diagnosis and caregiver report. Conditions associated with major mobility impairments (those with spina bifida or cerebral palsy, or those who reported “other physical disability”) were classified as physical disabilities. Caregivers additionally reported if their child had a diagnosis of ADHD in addition to their primary chronic condition.

Caregivers indicated the number of medicines taken by mouth per day and injections received per week by the child. We consolidated this information into a separate variable; the total number of medications and injections per week.

Caregivers also reported the number of times their child visited their primary care provider in a typical year and the number of ER visits in the past year. ER utilization was analyzed as a categorical variable (no ER visits or some ER visits, due to the broad range of ER visits among those who had them) while the primary care provider visit frequency was coded into three categories (one visit, two visits, three or more visits) due to the wide range of visits above three visits. All participants had at least one primary care visit per year.

Caregivers answered a modified version of the Zarit Burden Scale [18]. This 22-question tool measures caregiver burden and all questions are answered on a five-point Likert scale (ranging from “never” to “nearly always”). The adapted version refers to the patient as “the child for whom you’re caring” [19]. Higher total scores on this scale indicate higher burden of the caregiver with regards to caregiving for their child. The Zarit Scale has been used in caregivers of youth and young adults in previous studies [20,21].

We utilized three validated tools to measure self-management skills of the child. Child participants answered these questions independently and privately using the following two methods:

(1) Self-Efficacy Scale [22]: This scale is adapted from a scale pertaining to diabetes and measures self-efficacy using nine questions on a five-point Likert scale. Questions were modified to be usable with a variety of chronic illnesses. We have used this adapted version of the scale in previous studies [23,24]. The possible range of scores was 5–45, with higher scores indicating greater self-efficacy. This scale was originally developed for adolescents (ages 10–16 years).
(2) The STAR_x_ (Self-management and Transition to Adulthood with R_x_ = therapy) Survey [25]: This 18-question self-reported scale measures knowledge and self-management skills needed for children to successfully transition from pediatric to adult care. Individual items were scored on a five-point Likert scale. The possible range of scores was 0–90, with higher scores indicating greater transition readiness. The survey was developed with pilot testing in youth with chronic conditions (ages 8–25 years) [25].

### 2.4. Statistical Analysis

Three separate linear regression analyses were conducted. All models controlled for age, gender, race (white vs. non-white), and physical disability status (physical disability vs. no physical disability) of the child. Age, gender, and race are often associated with transition readiness and other variables in the study [26,27,28]. Controlling for age also allowed us to account for the likely increases in scores with age on the self-efficacy and transition readiness scales.

In order to preserve power, we decided to separate our independent variables a priori into three categories (disease characteristics, health care utilization and self-management). A separate regression was run for each of these three categories. The first regression model included disease characteristics consisting of the following independent variables: total number of medicines and injections per week; child ADHD diagnosis; and percentage of child’s life with the disease. The second model focused on healthcare utilization and included primary care provider and ER visits as independent variables. Lastly, the third model included independent variables related to self-management such as self-efficacy and transition readiness. The dependent variable was caregiver burden in all three analyses. All analyses were conducted in SPSS version 22 (IBM, Armonk, NY USA), and significance was determined at *p* < 0.05.

## 3. Results

We enrolled a sample of 160 caregiver-youth dyads (20% response rate). Because there were ten father or grandparent caregivers, we focused the rest of our analysis on mother-youth dyads, totaling 150 children and their mothers. The mean age of child participants was 12.23 (±2.5), with an age range of 6 to 16 years, and their mean age at diagnosis was 2.79 ± 3.7 years. Table 1 describes the characteristics of the sample.

The child participants took between 0 and 20 medicines by mouth per day (mean: 2.59 ± 3.3) and between 0 and 7 injections per week (mean: 0.79 ± 2.1), and had had their disease for an average of 77.2% of their lives. Self-reported ADHD was noted in 25.5% of youth. Participants visited the ER an average of 0.88 ± 1.9 times in the year prior to completion of the survey, and visited their primary care provider an average of 3.03 ± 3.6 times per year. Over half of the participants (64.8%) had no ER visits in the previous year. The mean score on the transition readiness survey was 54.9 ± 13.4 (range: 0–85), and the mean self-efficacy score was 56.1 ± 24.2 (range: 0–90).

There was wide variation in the burden as measured by the modified Zarit Burden Scale (range: 0–100), and the mean score was 52.55 ± 16.8. Mothers of white children reported significantly higher burden than mothers of non-white children (*p* = 0.017), with a mean burden score of 54.29 (±1.4) for mothers of white children and 46.39 (±3.5) for mothers of non-white children. There was a trend towards significance such that mothers of children with physical disabilities had higher burden (mean: 56.93 ± 3.0) than mothers of those without (mean: 50.96 ± 1.5) (*p* = 0.055).

Table 2, Table 3 and Table 4 show the results of the disease characteristics, healthcare utilization, and self-management models predicting burden.

The child’s number of medicines and injections taken and diagnosis of ADHD as a comorbidity predicted higher caregiver burden in the disease characteristics model (F_(7,141)_ = 3.02, *p* = 0.006). In the healthcare utilization model (F_(6,109)_ = 3.09, *p* = 0.008), more frequent primary care and ER visits predicted higher caregiver burden. Lastly, among the self-management factors (F_(6,116)_ = 2.86, *p* = 0.012), lower disease self-efficacy of the child predicted higher caregiver burden.

## 4. Discussion

Our study found that children’s number of medicines and injections, a diagnosis of ADHD in addition to the primary medical condition, frequent PCP and ER visits, and lower child self-efficacy were predictors of increased caregiver burden, while controlling for the child’s race, gender, and physical disability. Among the disease-related characteristics, the child’s diagnosis of ADHD as a comorbidity had the strongest association with burden. This may suggest that other accompanying disorders may have a greater impact on children than the complexity of the medical regimen or the length of the illness itself. Children with ADHD may display greater behavioral issues, and this is consistent with literature showing that behavioral problems may contribute to caregiver burden [29]. As no other mental disorders were included in the current study, it would be important for future studies to explore the impact of additional mental health disorders that frequently coexist among children with chronic conditions, such as depression and anxiety, as well as other comorbid developmental disabilities [30,31].

The association between the number of medicines and injections taken by the child per week and the mother’s burden may suggest that mothers are very involved in helping their children obtain or remember their medicines. The association between ER visits and burden may be because a higher number of health emergencies can contribute to stress and anxiety. However, contrary to our original hypothesis, more frequent PCP visits also predicted greater burden. This suggests that the overall frequency, rather than the type (preventative vs. emergency), of healthcare utilization contributes to caregiver burden. It is also important to note that health utilization may not be a causal factor, and it may be just as likely that the high healthcare utilization was caused by the mother’s burden and desire for help in taking care of the child in a stressful situation. Due to the diverse sample with several medical conditions represented, we were unable to have one measure of illness severity across all subjects in this study. However, a greater number of medicines and higher healthcare utilization may reflect a more severe illness, which may be one explanation for the higher burden.

Health-related self-efficacy is the child’s belief in his or her capacity to manage their disease. Our results indicated that mothers of children with greater self-efficacy had lower burden. This may be because children are more able to perform the tasks needed to take care of their health, lessening the duties/demands of the mother. Because we cannot determine causality from our results, an alternative explanation may be that children of mothers who are participating less in their care have to become more self-sufficient. Interventions and programs to improve patients’ health-related self-efficacy may help alleviate caregiver burden, which may be an important secondary outcome for such programs to evaluate.

The high education level of the mothers was a significant limitation in this sample, as this is not representative of the overall educational attainment of people in North Carolina, where only 27.8% of people over the age of 25 years have a Bachelor’s degree or higher [32]. Although the camp and transportation to it is free, Victory Junction has a high attendance of children who may have access to the internet or other resources to help them sign up for camp, which may explain the high education level of their parents. Our sample also had a very high number of white families participate as compared to non-white families, and mothers of white children reported higher burden than those of non-white children, an association that has been well-characterized in the literature [26,28,33,34]. The sample lacked representation of fathers and grandparents, which led us to eliminate them from the analysis and focus on mothers. However, because literature on father and grandparent caregivers is lacking, future studies should attempt to increase representation of them when recruiting participants.

Additionally, because the survey was distributed online, data on those without access to the internet was not collected, which may have contributed to the low response rate (20%). Due to the online format, we were unable to ascertain that children completed the survey independently and privately. In the future, such a study would benefit from being conducted in-person in order to monitor the completion of the survey by the child participants. However, an advantage to the online distribution was the lack of bias due to social desirability, additionally strengthening our self-reported measures. Our study was additionally limited by reliance on self-report for health care utilization, a cross-sectional design, and limited sample size. The low R^2^ values for our models suggest that there are several other characteristics that contribute to caregiver burden that were not explored in this study. Future studies should examine the relationship of other dimensions and measures of disease characteristics, health utilization, and self-management to caregiver burden.

The variety in geographic backgrounds and diagnoses of the participants was a significant strength in our study. This allowed us to have a broad distribution for characteristics such as the number of medicines and injections taken and healthcare utilization of the child. We were therefore able to identify predictors of caregiver burden that are common across many different diagnoses and levels of disease severity. Another strength of this study is the use of a validated tool to measure caregiver burden. The Zarit Burden Interview has been modified and used in both pediatric and elderly populations, and its adaptations have been used in different countries and languages [35]. Therefore, there is a wide variety of additional literature highlighting the consequences and implications of caregiver burden as measured by this scale. However, because this scale has not been used in patients with some of the chronic diagnoses represented in this study, we were unable to use published average values for the Zarit Burden score to compare our results with those in the literature.

Burden among caregivers can have very negative consequences. Gallagher and colleagues found that caregiver burden, as measured by the same scale used in this study, was a strong predictor of depression and anxiety among parents of children with intellectual disability [36]. In addition to psychological consequences, caregivers with more burden also experience impaired health habits [37]. Caregivers may neglect their own health needs, forgoing preventative physician’s visits, healthy eating, and exercise, all which may lead to poor health [38]. Our results will allow providers to take into consideration some child-related risk factors for caregiver burden—more medications, ADHD as a comorbidity, high frequency of healthcare utilization, and low self-efficacy. By identifying mothers that are at high risk for burden based on these four factors, healthcare providers of both the child and the mother could make recommendations for managing or minimizing it.

The predictors identified could be targets for interventions to reduce caregiver burden. Present methods to reduce caregiver burden include interventions to increase disease knowledge, psychoeducational programs, and educational interventions in self-care, stress management, and communication skills [39,40]. In addition, interventions for caregivers of elderly people may be adapted and used for mothers of children with chronic illnesses and disabilities. For example, writing exercises and mindfulness-based stress reduction have been shown to reduce stress among caregivers [41,42]. Intervention strategies that go beyond the caregiver level may also be effective in this population. Our study found that child self-efficacy was a predictor of lower caregiver burden, and therefore interventions to improve patient self-efficacy may also help to alleviate burden of their caregiver. Data on parents of children is needed before any of these interventions can be recommended, and additional studies are needed to examine if providing families with interventions can lead to positive outcomes in both the caregiver and the child.

## Figures and Tables

**Table 1:** Child and caregiver characteristics (*n* = 150).

Child Characteristics	*n*	%
**Sex**		
Male	79	52.7
Female	71	47.3
**Race**		
White	117	78.0
Non-white	33	22.0
**Insurance**		
Private	99	66.0
Public	44	29.3
No insurance	7	4.7
**Primary Diagnosis**		
Cerebral palsy	26	17.3
Diabetes	21	14.0
Gastrointestinal diseases	13	8.7
Heart diseases	12	8.0
Sickle cell anemia	12	8.0
Cancer	9	6.0
Kidney disease	9	6.0
Other physical disability	9	6.0
Down syndrome	7	4.7
Neurological conditions	7	4.7
Craniofacial anomalies	6	4.0
Spina bifida	5	3.3
Lung diseases	5	3.3
Other genetic conditions	4	2.7
Bleeding disorders	3	2.0
Skin diseases	2	1.3
**Caregiver (mothers’) Characteristics**		
**Race**		
White	126	84.0
Non -white	24	16.0
**Education**		
Did not complete high school	3	2.0
High school or GED	8	5.3
Some college	31	20.7
Completed college	67	44.7
Some graduate school	5	3.3
Completed graduate school	36	24.0

GED: General Education Development

**Table 2:** Predicting caregiver burden by disease-related characteristics (Model R^2^ = 0.130).

Independent Variables		Unstandardized Coefficients		Standardized Coefficients	*t*	Sig.
		B	Std. Error	Beta		
Control variables	White race	5.761	3.171	0.145	1.817	0.071
	Physical disability	5.426	3.192	0.148	1.700	0.091
	Age	0.399	0.523	0.062	0.762	0.447
	Female gender	0.885	2.596	0.027	0.341	0.734
Disease-related characteristics	Total number of medicines or injections per week	0.114	0.057	0.161	2.005	0.047
	ADHD Diagnosis	8.059	2.991	0.216	2.695	0.008
	Percent of life with disease	−1.405	4.611	−0.026	−0.305	0.761

ADHD: attention-deficit/hyperactivity disorder; Std. Error: Standard Error; Sig: Significance.

**Table 3:** Predicting caregiver burden by healthcare utilization (Model R^2^ = 0.145).

Independent Variables		Unstandardized Coefficients		Standardized Coefficients	*t*	Sig.
		B	Std. Error	Beta		
Control variables	White race	6.999	3.602	0.178	1.943	0.055
	Physical disability	4.191	3.401	0.115	1.233	0.22
	Age	0.259	0.611	0.04	0.424	0.672
	Female gender	2.12	3.022	0.064	0.701	0.485
Healthcare utilization	Visits to primary care provider (PCP) in typical year	4.109	1.822	0.209	2.256	0.026
	Emergency room (ER) visits past year	6.883	3.281	0.197	2.098	0.038

**Table 4:** Predicting caregiving burden by self-management skills (Model R^2^ = 0.129)

Independent Variables		Unstandardized Coefficients		Standardized Coefficients	*t*	Sig.
		B	Std. Error	Beta		
Control variables	White race	6.789	3.733	0.162	1.819	0.072
	Physical disability	5.537	4.038	0.13	1.371	0.173
	Age	0.66	0.624	0.097	1.058	0.292
	Female gender	1.114	3.166	0.032	0.352	0.725
Self-management skills	Self-efficacy	−0.19	0.088	−0.246	−2.155	0.033
	Transition readiness	0.013	0.167	0.01	0.08	0.936
